# Characterization of a the *Plasmodium falciparum* Hsp70-Hsp90 organising protein

**DOI:** 10.1186/1475-2875-9-S2-P11

**Published:** 2010-10-20

**Authors:** Grace Gitau, Addmore Shonhai

**Affiliations:** 1 Department of Biochemistry & Microbiology, Zululand University, Kwadlangezwa, 3886, South Africa

## Background

Heat shock proteins act as molecular chaperones, regulating protein folding in the cell. In *Plasmodium falciparum,* heat shock proteins do not only facilitate protein folding but are also implicated in trafficking of proteins within the parasite and to the erythrocyte surface. Thus heat shock proteins are thought to augment parasite infectivity, and are also indirectly involved in conferring drug resistance to the parasite. *Plasmodium falciparum* possesses heat shock protein 70 (PfHsp70-1) and heat shock protein 90 (PfHsp90) and both chaperones are essential for parasite survival. It is known that some proteins, especially those involved in signal transduction, require both heat shock protein 70 (Hsp70) and Hsp90 for them to be folded into functional forms. The Hsp70-Hsp90 functional partnership has been well characterized in mammalian systems and is coordinated by an adaptor protein, Hsp70-Hsp90 organising protein (Hop). It has been proposed that PfHsp70-1 and PfHsp90 occur in a chaperone functional network in the malaria parasite [1,2]. Hop possesses three TPR domains - TPR1 domain, located in the amino terminus, TPR2A (located in the middle) and the C-terminal, TPR2B domain. Both Hsp70 and Hsp90 are thought to interact with Hop via their C- terminal EEVM motifs. Hop interacts with Hsp70 via the TPR1 domain, and binds Hsp90 via the TPR2A domain. There is evidence suggesting that the PfHsp70-1/PfHsp90 pathway could be an ideal drug target [2]. In this study, we analysed structure-function studies on a putative Hop homologue from *P. falciparum* (PfHop; PF14_0324).

## Materials and methods

Protein sequence were downloaded from from Plasmodband NCBI and aligned by to identify structural motifs important for interaction of PfHop with both Hsp70 and Hsp90 proteins. Furthermore, we isolated and PCR amplified the DNA segment encoding the putative PfHop towards heterologous overexpression and purification of the protein. We also wish to conduct immunoflourescence and immunoprecipitation assays to establish the expression and localisation profiles of PfHop at the various life stages of the *P. falciparum* parasite.

## Conclusion

The putative PfHop displays conserved structural motifs known to be important for interaction with Hsp70 and Hsp90 proteins, suggesting that PfHop is a potential adaptor protein that functionally links PfHsp70-1 and PfHsp90. We wish to conduct biochemical assays to further ascertain the role of PfHop in the PfHsp70-1/PfHsp90 pathway.

## References

[B1] Banumathy G Singh V Tatu U Host chaperones are recruited in membrane-bound complexes by *Plasmodium falciparum* J Biol Chem 2002 277 3902 3912 10.1074/jbc.M110513200 11711553

[B2] Shonhai A Plasmodial heat shock proteins: targets for chemotherapy FEMS Immunol Med Microbiol 2010 58 61 74 10.1111/j.1574-695X.2009.00639.x 20041948

